# Transthoracic minimally invasive closure for the treatment of arch penetrating aortic ulcer: a case report

**DOI:** 10.1186/s13019-021-01659-9

**Published:** 2021-10-09

**Authors:** Shixiong Wang, Debin Liu, Yongnan Li, Bingren Gao

**Affiliations:** Department of Cardiac Surgery, Lanzhou University Second Hospital, No. 82 Cuiyingmen, Lanzhou, 730030 Gansu People’s Republic of China

**Keywords:** Penetrating aortic ulcer, Transthoracic, Closure

## Abstract

Penetrating aortic ulcer (PAU) is one of the three subtypes of acute aortic syndrome. PAUs occur at any point throughout the aorta, most commonly in the descending thoracic aorta and less frequently in the aortic arch. Open surgical repair and total/hybrid endovascular repair are currently available to treat aortic arch PAUs. Herein, we present a patient with aortic arch PAU who underwent transthoracic minimally invasive closure, which is a novel method for the treatment of PAU. We describe a 52-year old Asian man who presented with sudden chest and back pain for 8 h. Computed tomography angiography showed that the PAU occurred in the aortic arch and had a diameter of 16 mm and a depth of 6 mm. The opening was successfully closed via transthoracic minimally invasive closure with an atrial septal defect occluder.

## Background

Penetrating aortic ulcer (PAU) is one of the three subtypes of acute aortic syndrome (AAS) and is defined as ulceration of an aortic atherosclerotic plaque penetrating the internal elastic lamina into the media and often associated with a variable degree of intramural haematoma (IMH) formation [1, 2]. PAUs may account for 2.3–7.6% of cases of AAS [3–5], and the incidence of PAU significantly increased, from 0.6 to 2.6 per 100,000 person-years, from 1995 to 2015 [6]. Typically, PAUs develop as isolated lesions and are mainly located in the descending thoracic aorta [5], with only 7% of PAUs occurring in the aortic arch [7]. Open surgical repair and total/hybrid endovascular repair are currently available to treat aortic arch PAUs. Herein, we report the first case of an aortic arch PAU closed via transthoracic minimally invasive closure with an atrial septal defect (ASD) occluder.

## Case presentation

**Fig. 1:**
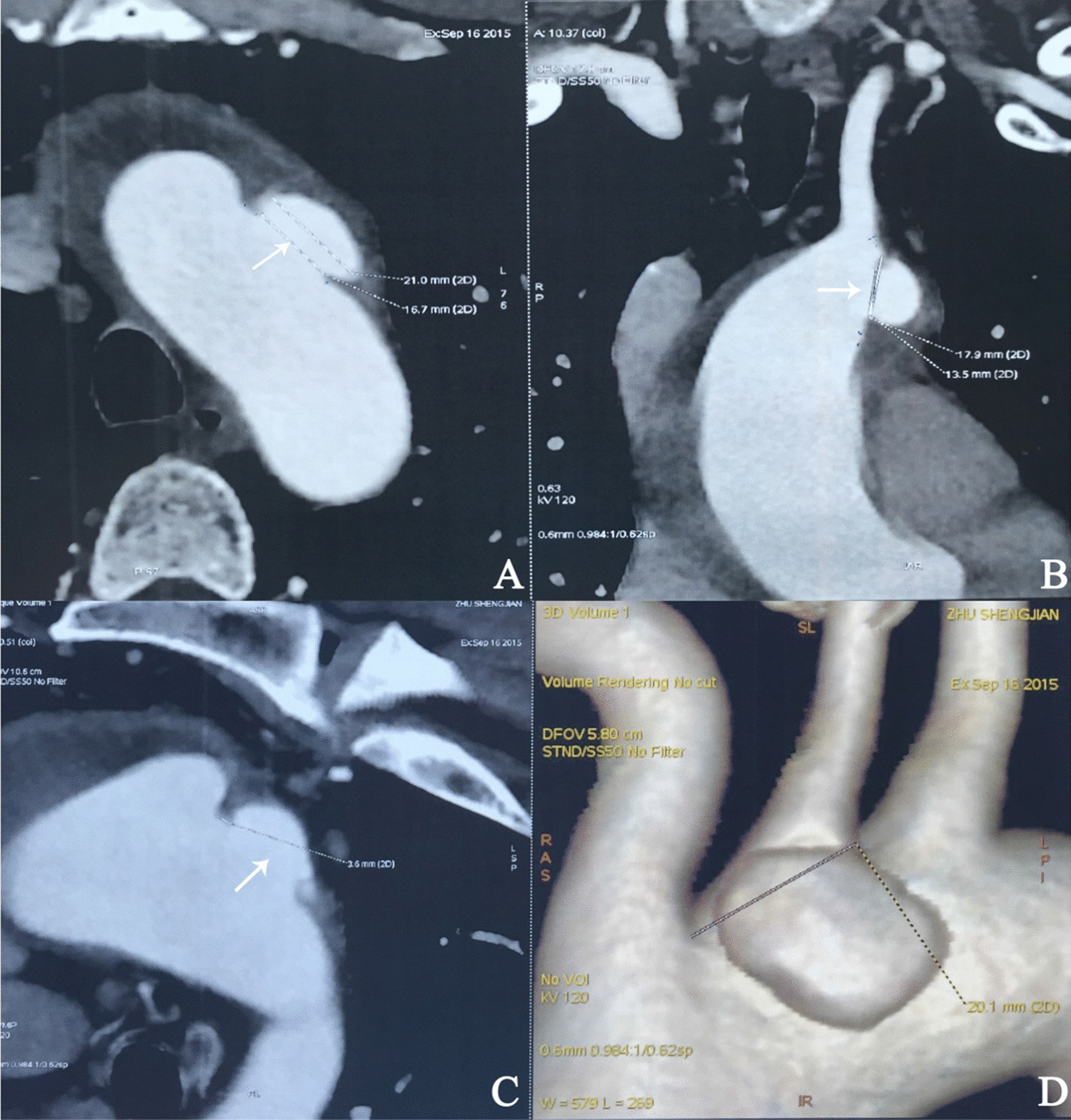
Preoperative imaging. Computed tomographic angiography (CTA) showing a penetrating aortic ulcer (PAU) that occurred in the aortic arch, with an intramural haematoma (IMH) compromising the ascending aorta and aortic arch

A 52-year old Asian man was admitted to our department with sudden chest and back pain for 8 h. The patient had a history of hypertension, diabetes, coronary atherosclerotic heart disease, cerebral embolism and chronic obstructive pulmonary disease. An aortic computed tomography angiography (CTA) examination upon admission showed an IMH compromising the ascending aorta and aortic arch. The PAU occurred in the aortic arch and had a diameter of 16 mm and a depth of 6 mm (Fig. 1), the diameter of ascending aorta was 38 mm. Echocardiography showed no dilated ascending aorta, no aortic valve regurgitation, and normal heart function. He was maintained on an adequate pain control medication (intravenous opiate analgesia), with meticulous blood pressure control (< 120/80 mmHg) and heart rate control (< 60 b.p.m.) after the admission.

**Fig. 2:**
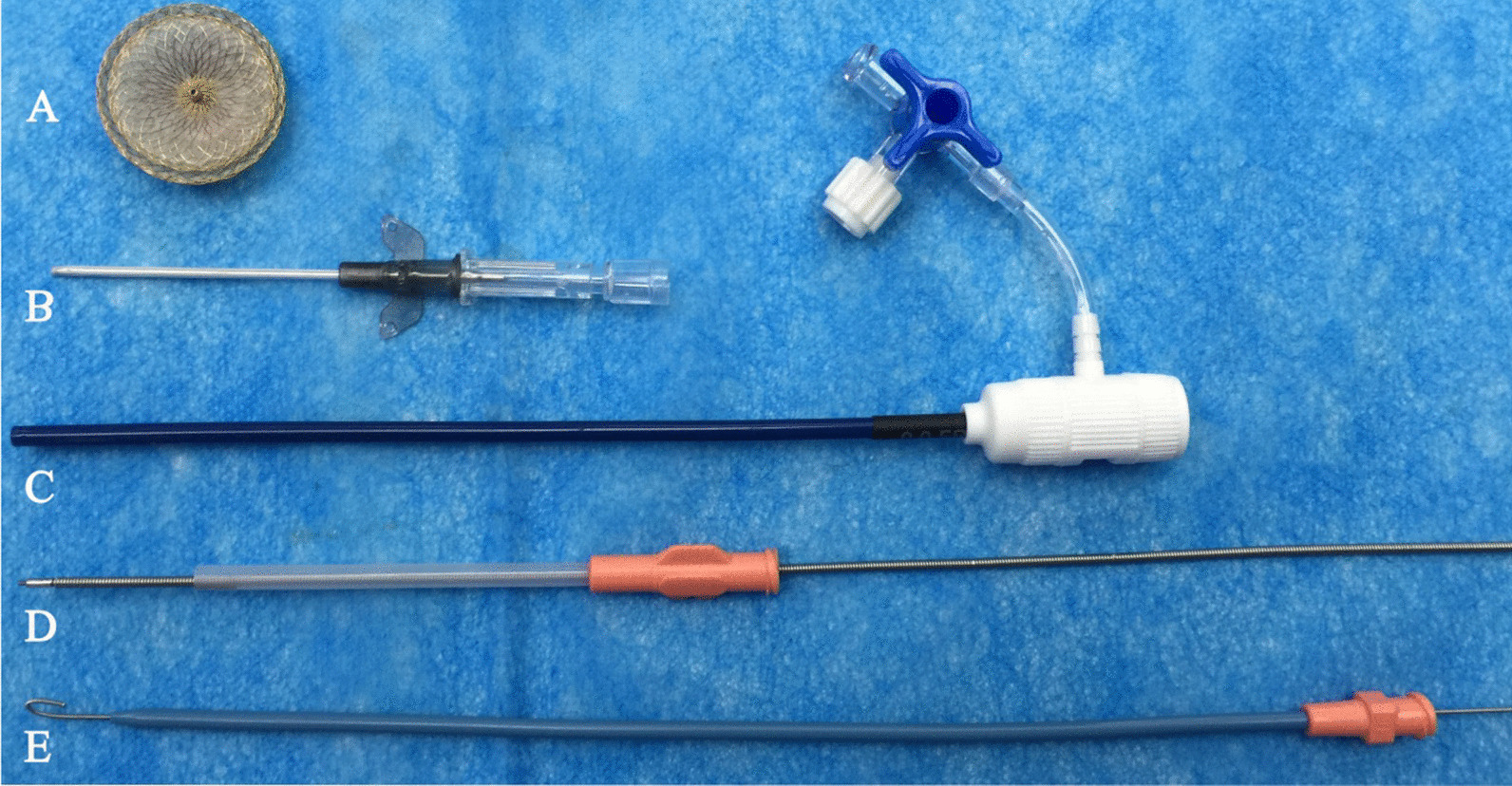
Delivery system: **A** ASD occluder. **B** 16-G trocar. **C** Delivery sheath. **D** Loading sheath. **E** Guide sheath and guidewire

We explained all treatment options to the patient, including preoperative preparation, the procedure and complications, as aortic arch replacement involves high risk and great trauma. Furthermore, he wanted to avoid the risk of reoperation as the total/hybrid endovascular repair is more complicated. After providing informed consent, the patient agreed to transthoracic minimally invasive closure of the PAU (Fig. 2).

**Fig. 3:**
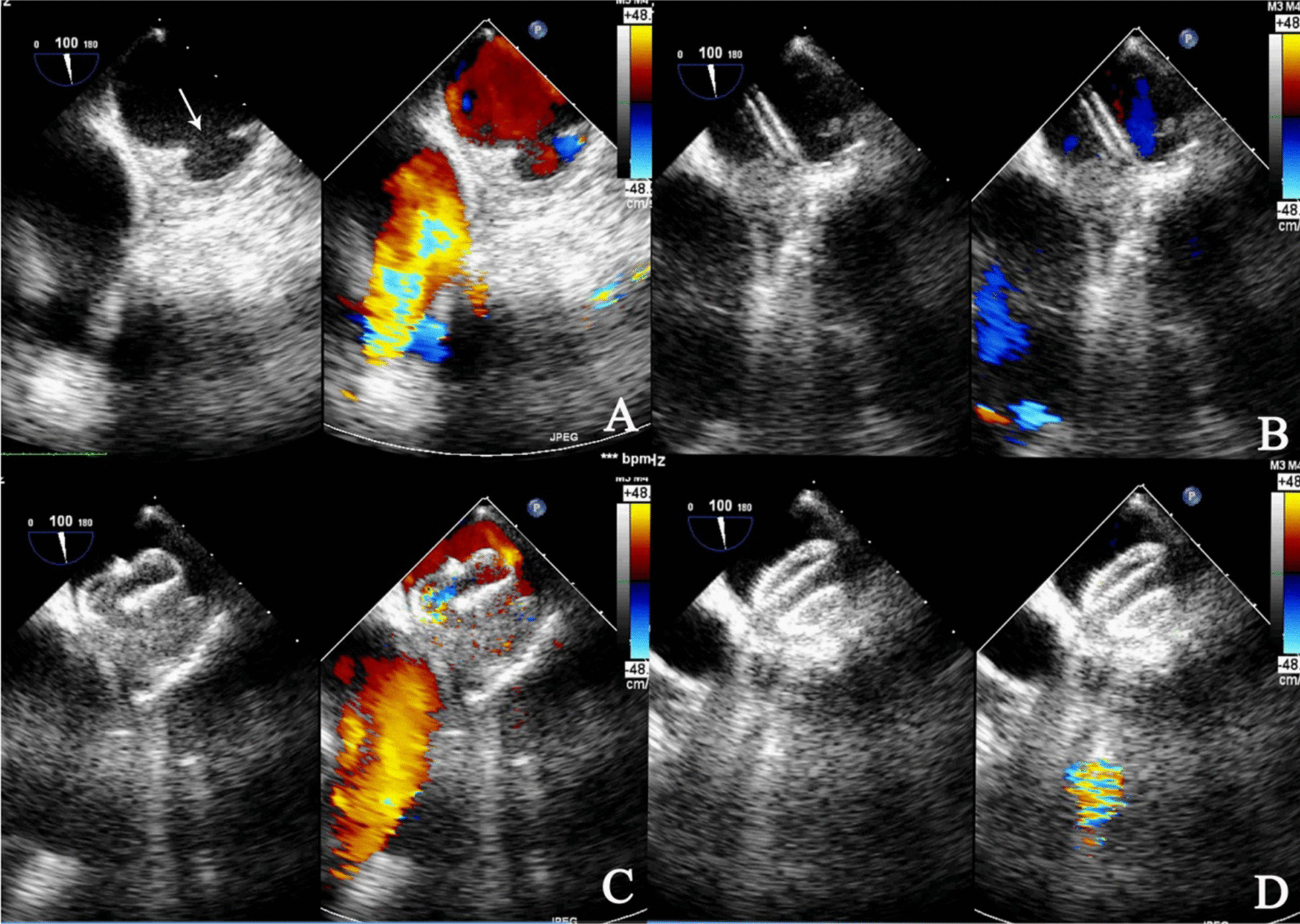
Sequential TEE and illustration images of transthoracic minimally invasive closure of an arch penetrating aortic ulcer (PAU). **A** TEE shows the PAU. **B** A delivery sheath was introduced through the defect and positioned with its tip in the ascending aorta. **C** Occluding device was delivered through the prepared loading sheath and delivery sheath. The left ventricular disc was deployed first by pushing the cable forward and then rotated until the disc advanced to the opposite side of the aorta thoracica. **D** An ASD occluder was advanced along the sheath. When the true lumen-to-false lumen shunt disappeared, the device was deployed. There was no significant residual shunt

After general anaesthesia and intubation, the patient was placed in a supine position. A transoesophageal echocardiography (TEE) probe was inserted to monitor the procedure. The shape, location and maximal diameter of the PAU were measured by TEE (Fig. 3). After TEE assessment, the proper device was selected.

The incision was made from the superior sternal fossa to the third intercostal space, with a length of approximately 5 cm. The sternum was cut in an inverted "L" shape, the surrounding tissues of the aortic arch were separated, and the aortic arch was exposed. To determine the puncture site, the aortic arch was slightly palpated under the guidance of TEE to locate the area of the maximal thrill, corresponding to the location of the PAU rupture. A purse-string suture was placed at this location.

**Fig. 4:**
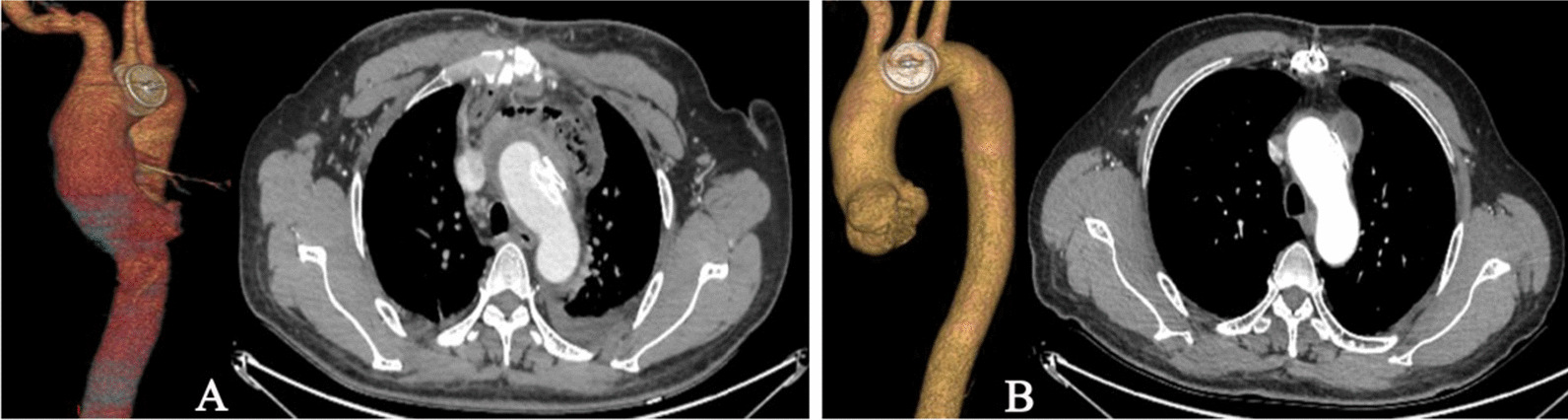
Postoperative imaging. **A** 1 month after discharge. **B** 7 months after discharge. The CTA images show that the occluder was placed well and had a normal morphology, without aortic dissection

After anticoagulation with heparin (1.0 mg/kg), the centre of the purse was punctured with a trocar, and as arterial blood spurted from the trocar, the needle was removed, and a hyperechogenic guidewire was inserted. Next, the guidewire was placed into the true lumen (TL) through the PAU rupture. Then, a 9-Fr delivery sheath (Lifetech Scientific Co., Ltd., Shenzhen, China) was moved into the TL along the guidewire. An ASD occluder with a waist diameter of 16 mm was implanted along the sheath. The first disc was opened in the TL and was pulled back to be anchored at the rupture site; then another disc was opened in the false lumen (FL). When the TL-to-FL shunt disappeared, the device stability was ascertained by a push–pull manoeuvre, and the device was released upon satisfactory assessment (Fig. 3). The whole procedure was guided and monitored by TEE. After detaching the device from the delivery cable, TEE was repeated to evaluate the position of the occluder, residual shunt and aortic dissection. The patient recovered well and was discharged a few days later, with no further complications. The patient received aspirin (100 mg per day) for a six-month period. Aortic CTA was performed at intervals of 1, 3 and 7 months after discharge (Fig. 4). The results were satisfactory, no residual shunt, aortic dissection and the diameter of ascending aorta was still 38 mm. There was no residual shunt, stroke, aortic dissection and other significant complications during 57 months of follow-up.

The procedure was uncomplicated, and the patient remained well at a 5-year clinical follow-up.

## Discussion

The natural history of PAU is not completely clear; it is potentially associated with IMH and aortic dissection and leads to aortic wall rupture [7]. Up to 38% of PAUs have an abrupt onset with rupture; those presenting with pain have been associated with morphologic progression and complications [8]. It is thought that patients with a PAU that initially measures > 20 mm in diameter or > 10 mm in depth have a high risk of disease progression [7]. For these reasons, the general consensus is to consider surgery for Type A and all complicated PAUs [2, 9, 10].

The treatment of PAU remains controversial. Although open surgical repair is still the gold-standard treatment for aortic arch diseases, primarily for aneurysms and dissections, open surgery has been associated with high morbidity and mortality rates (9–38%) [11, 12]. With the improvement of biomaterial products and the development of surgical technologies, new devices and techniques for total/hybrid endovascular repair are currently available to treat aortic arch PAUs [13–15]. PAUs of the arch have been considered adequate anatomic targets for thoracic endovascular aortic repair (TEVAR), and thoracic PAUs have been defined as an ideal surgical indication for TEVAR [9, 15]. However, PAUs developed in the arch are the most challenging to treat endovascularly, with a high mortality rate and arch and access vessel complications, due to the variability of the anatomy of the arch and its branches [16]. Kleisli and et al. [17] reported closure of a PAU of the descending aorta with an Amplatzer Occluder via percutaneous femoral access in 2009, the 12-month follow-up was also satisfactory. It was may be a viable alternative for patients who are not candidates for endovascular repair secondary to poor vascular access or large aortic diameters. But the arch of the aorta is not so easy to manipulate.

To overcome these problems, we applied a novel method of transthoracic minimally invasive closure with an ASD occluder to close the rupture of the PAU under the guidance of TEE. The early and follow-up outcomes were satisfactory. In our procedure, a small incision in the upper sternum could reduce the trauma and keep the stability of the sternum. Transthoracic closure avoids the limitations of vascular conditions, simplifies the delivery path and reduces the damage from radiation. In addition, we can easily switch to the conventional sternotomy treatment if the closure fails. Apart from the feasibility of the procedure, caution must be used to exclude any significant aortic valve regurgitation or residual shunt, which may be associated with haemolysis due to a high-pressure flow or infective endocarditis. Nevertheless, long-term follow-up and increased clinical experience is needed to prove that it is a safe procedure.

## Conclusions

Under the guidance of TEE, transthoracic minimally invasive closure of PAU rupture may be a safe, efficient and innovative treatment method for suitable patients, allowing various complications caused by complex surgery to be avoided, trauma to be reduced, and the length of hospitalization to be shortened.

## Abbreviations

PAU: Penetrating aortic ulcer
IMH: Intramural haematoma
CTA: Computed tomography angiography
TEE: Transoesophageal echocardiography
ASD: Atrial septal defect
